# Canadian National Pancreas Conference 2023: A Review of Multidisciplinary Engagement in Pancreatic Cancer Care

**DOI:** 10.3390/curroncol31100461

**Published:** 2024-10-16

**Authors:** Jessica L. Nickerson, Chloe Cyr, Riley J. Arseneau, Stacey N. Lee, Stefanie Condon-Oldreive, George Zogopoulos, Keith Roberts, Christina A. Kim, Sylvia S. W. Ng, Masoom Haider, Eva Villalba, Leah Stephenson, Erica Tsang, Brent Johnston, Boris Gala-Lopez, Valerie Cooper, Breffni Hannon, Anne Gangloff, Sharlene Gill, Filomena Servidio-Italiano, Ravi Ramjeesingh

**Affiliations:** 1 Allumiqs Corporation, Halifax, NS B3H 0A8, Canada; jessica@craigscause.ca; 2 Craig’s Cause Pancreatic Cancer Society, Halifax, NS B3K 5M3, Canada; chloe.cyr@dal.ca (C.C.); rl686785@dal.ca (R.J.A.); stacey.lee@dal.ca (S.N.L.); stefanie@craigscause.ca (S.C.-O.); 3 Department of Kinesiology, Dalhousie University, Halifax, NS B3H 4R2, Canada; 4 Beatrice Hunter Cancer Research Institute, Halifax, NS B3H 0A2, Canada; 5 Department of Pathology, Dalhousie University, Halifax, NS B3H 4R2, Canada; 6 Department of Microbiology and Immunology, Dalhousie University, Halifax, NS B3H 4R2, Canada; brent.johnston@dal.ca; 7 McGill University Health Centre, Montreal, QC H4A 3J1, Canada; george.zogopoulos@mcgill.ca; 8 Institute of Immunology and Immunotherapy, University of Birmingham, Birmingham B15 2TT, UK; keith.roberts@uhb.nhs.uk; 9 Paul Albrechtsen Research Institute CancerCare Manitoba, Winnipeg, MB R3E 0V9, Canada; ckim3@cancercare.mb.ca; 10 Section of Medical Oncology and Hematology, Department of Internal Medicine, Rady Faculty of Health Sciences, University of Manitoba, Winnipeg, MB R3E 0W2, Canada; sylvia.ng@sunnybrook.ca; 11 Department of Radiation Oncology, Sunnybrook Health Sciences Centre, Toronto, ON M4N 3M5, Canada; 12 Joint Department of Medical Imaging, Sinai Health System, Toronto, ON M5G 1X6, Canada; mahaider@radfiler.com; 13 Quebec Cancer Coalition, Saint-Lambert, QC J4P 2J7, Canada; evav@coalitioncancer.com; 14 All.Can, Pentincton, BC V2A 0B2, Canada; leah.allcancanada@saveyourskin.ca; 15 Department of Medicine, Princess Margaret Cancer Centre, Toronto, ON M5G 2C4, Canada; erica.tsang@uhn.ca; 16 Department of Surgery, Dalhousie University, Halifax, NS B3H 2Y9, Canada; b.gala-lopez@dal.ca; 17 South East Local Health Integration Network, Belleville, ON K8N 5K3, Canada; valerie.cooper@hccontario.ca; 18 Department of Supportive Care, Princess Margaret Cancer Centre, Toronto, ON M5G 2C4, Canada; breffni.hannon@uhn.ca; 19 Faculty of Medicine, Laval University, Quebec, QC G1V 0A6, Canada; anne.gangloff.1@ulaval.ca; 20 BC Cancer, Vancouver, BC V5Z 4E6, Canada; sgill@bccancer.bc.ca; 21 Colorectal Cancer Resource and Action Network (CCRAN), Toronto, ON M4W 3E2, Canada; filomena.s@ccran.org; 22 Division of Medical Oncology, Dalhousie University, Halifax, NS B3H 2Y9, Canada

**Keywords:** pancreatic cancer, collaborative care, multidisciplinary meeting

## Abstract

Pancreatic cancer is a complex malignancy associated with poor prognosis and high symptom burden. Optimal patient care relies on the integration of various sectors in the healthcare field as well as innovation through research. The Canadian National Pancreas Conference (NPC) was co-organized and hosted by Craig’s Cause Pancreatic Cancer Society and The Royal College of Physicians and Surgeons in November 2023 in Montreal, Canada. The conference sought to bridge the gap between Canadian healthcare providers and researchers who share the common goal of improving the prognosis, quality of life, and survival for patients with pancreatic cancer. The accredited event featured discussion topics including diagnosis and screening, value-based and palliative care, pancreatic enzyme replacement therapy, cancer-reducing treatment, and an overview of the current management landscape. The present article reviews the NPC sessions and discusses the presented content with respect to the current literature.

## 1. Introduction

Pancreatic adenocarcinoma (PC) is the eleventh most diagnosed cancer in Canada, but is the third leading cause of cancer-related death with a five-year survival rate of just 10% [1]. The high mortality rate associated with PC is largely attributed to a lack of screening technology, non-specific symptom presentation, consequential late diagnosis, and variable treatment response [2]. Compared to the improvements in overall survival rates with other major cancers (e.g., breast, lung), there has been relatively less impactful improvement in PC survival [3]. Although surgical resection can be curative, only 20% of patients are diagnosed with resectable disease (stage I or II) [4]. Despite decades of research, chemotherapy and radiotherapy approaches have only led to incremental improvements in survival rates, largely due to treatment resistance [5]. Regardless of a patient’s candidacy for curative-intent resection, palliative care approaches have been proven to reduce symptom burden and improve quality of life [6].

Successful and widespread improvement in PC clinical outcomes relies on a collaboration among experts across healthcare and research fields. For example, advances in imaging, surgery, systemic therapy, targeted medicine, and integrated healthcare have improved patient outcomes [5]. This reliance on collaboration between healthcare sectors and researchers necessitates engagement opportunities, where individuals can gain an understanding of how their own role fits within a complex network of experts who are also working to improve the outcomes of PC. The 2023 National Pancreas Conference (NPC)—hosted by Craig’s Cause Pancreatic Cancer Society in Montreal, Quebec—was the first in-person NPC event following a virtual pilot conference in 2021. The newly established NPC series represents the only scientific conference in Canada for PC. The event was co-developed by the Canadian Association of General Surgeons, and accredited through the Royal College of Physicians and Surgeons, enabling healthcare provider (HCP) participants to earn up to 7.5 Section 1 Maintenance of Certification credits. The conference brought together healthcare experts, researchers, and cancer-care advocates from across Canada to discuss recent advances in PC treatment, management, and promising research. Topics were selected based on the results of a thorough needs assessment (Supplement File S1), which highlighted gaps and areas of interest among PC experts and care providers. Key learning objectives were defined for each session (Supplement File S2). The conference featured a keynote session on pancreatic enzyme replacement therapy (PERT), which highlighted the often-underestimated role of nutrition in improving patient outcomes. Following this session, the novel PERT Calculator application was introduced as a tool to enable tailored dosing of PERT based on specific input food combinations. Subsequent sessions of the conference featured panel discussions on screening, diagnosis, treatment, integrative practice, and clinical trials. Poster sessions and social events provided attendees with engaging opportunities to connect and collaborate. To further facilitate ongoing and future collaborative and multidisciplinary efforts, research grants were presented to faculty and graduate students, based on submitted proposals. The present review summarizes key themes and calls to action concluded from the 2023 NPC.

## 2. Diagnosis and Screening

PC is a high-mortality cancer, with a five-year survival rate of just 10% [1]. Low survival in PC is largely attributed to treatment resistance and late-stage diagnosis, with 80% of patients being diagnosed at stages III-IV [4,7,8]. For the <20% of PC patients who are diagnosed early (stage I-II), the average five-year survival rate improves to 44% due to their candidacy for a curative-intent pancreatectomy [9,10]. Overall, the ambiguous and late-presenting symptoms associated with PC warrant screening of high-risk populations to drive detection of early-stage PCs and high-grade precursor lesions. Screening is not recommended for the general population considering the relatively low lifetime risk for developing PC (1.7%), and the limitations of the current imaging modalities [11]. However, within high-risk populations (lifetime risk of 5% or more), including those with familial PC or mutations in a PC predisposition gene (e.g., *BRCA2*), new onset diabetes, and chronic pancreatitis, screening is proposed to improve patient outcomes while remaining cost effective [11,12,13].

The National Comprehensive Cancer Network has recommended germline genetic testing to PC patients across all disease stages [14]. Germline testing can identify hereditary predisposition syndromes, whereas somatic testing can only determine genetic alterations within tumor cells. Identifying a germline mutation in a patient with PC can help with targeted therapy decisions (i.e., patients with germline mutations in genes associated with homology recombination or mismatch DNA repair deficiency) [15]. A positive germline mutation result can also trigger cascade testing among their relatives for prevention and early detection of cancers associated with these mutations, including enrollment in PC screening programs. Despite the advantages of germline testing, equitable access to testing for patients diagnosed with PC remains a challenge [16]. Specifically, the accessibility of germline testing varies across Canadian healthcare institutions, and several factors (i.e., insurance concerns, logistics/travel burden, long wait times etc.) have been identified as barriers to care [17]. To improve testing uptake and timely return of results, point-of-care germline testing education and biospecimen collection have been integrated within ambulatory oncology clinics [18].

The PRECEDE consortium [19] is a multinational effort established to facilitate the expansion of PC screening programs with infrastructure to conduct surveillance of high-risk individuals in a research setting. Canadian research partners have collaborated in this work by sharing longitudinally-collected clinical and imaging data as well as biospecimens. PRECEDE’s overarching goal is to drive early detection of PC, and improve overall survival. The Canadian PRECEDE team is working to establish a liquid-biopsy screening program, generate organoid cell models, and screen drug libraries based on patient-specific genomic sequencing.

Other screening tools (i.e., MRI/MR-cholangiopancreatography (MRI/MRCP) and endoscopic ultrasound) can also be used for early detection in high-risk individuals. Outcomes from the Cancer of Pancreas Screening-5 (CAPS5) Study revealed that approximately 80% of participants in the screening imaging exam program were diagnosed with stage I disease, which is highly encouraging compared to the 5% standard [11]. However, individuals diagnosed with advanced-stage PC continue to have poor long-term survival and treatment outcomes. While the CAPS5 results are encouraging, they also highlight the need to refine screening strategies for high-risk individuals. Parallel development of predictive liquid-biopsy biomarkers (e.g., circulating tumor cells, circulating tumor DNA, non-coding RNAs) may help refine imaging intervals, with stratification of patients that merit closer imaging surveillance [20]. For example, given the prevalence of pancreatic cysts in high-risk individuals, biomarkers may serve as an effective screening tool for PC [21]. Implementation of molecular testing for detection of genetic alterations in endoscopically sampled fluid collections from evolving pancreatic cysts may help better identify high-grade precursor cystic lesions for early surgical resection in at-risk populations [3,21].

Imaging and radiomics are also important tools for early detection and monitoring in high-risk patients. Through scoring preoperative computed tomography (CT) scans, effective patient stratification based on a radiomic biomarker (Rad-score) has been established [22]. This score correlates with both patient survival and transcriptional subtype. Additionally, opportunistic screening (capitalizing on otherwise prescribed imaging for the screening of PC) has been recognized as an exciting avenue for early detection. Using radiomic machine-learning, early work has shown that PC is detected by the model for up to one year sooner than it was clinically diagnosed. [23]. While preliminary, this work highlights the potential of radiomics to refine less invasive diagnostic strategies.

### Calls to Action

- Improve germline testing for patients diagnosed with PC, with the implementation of oncology clinic-based point-of-care testing and timely return of results for treatment decisions.
- Improve cascade testing in families that harbor a predisposing germline mutation and expand screening opportunities for high-risk individuals across Canada, preferably through an integrated clinical and research program such as PRECEDE.
- Continue to optimize machine-learning models for improved detection of elusive early malignant and high-risk precursor lesions.

## 3. Integrated Value-Based Healthcare

Despite advances in medical and clinical research, oncology health inequities persist throughout Canada [24]. Systematic review and meta-analysis have identified considerable socioeconomic and racial disparities in timely diagnosis and access to treatment in PC globally, including in Canadian centers [25]. Value-based healthcare approaches may be key to improving diagnostic pathways and patient outcomes. “Value” is defined as the improvement in a patient’s health outcome compared to the cost of achieving that improvement [26,27]. Rather than pre-determining a patient’s course of treatment based exclusively in clinical indicators, a value-based approach considers the goals and preferences of the individual when defining a “good outcome”. This approach aims to empower patients by increasing their control over their healthcare decisions, maintaining autonomy, and emphasizing the role of quality of life in PC care.

Optimal value-based healthcare requires collaboration among HCPs as described by Porter & Teisberg [27]. In their book, *Redefining Healthcare*, the authors propose a shift towards more efficient prevention, diagnosis, and treatment of disease. Specifically, strategies to simultaneously improve patient experience of care, health of populations, cost of care, and clinician experience have been called for [26]. Such a shift would optimize patient care and outcomes per dollar invested, as opposed to simply reducing costs [28]. Value-based healthcare prioritizes outcomes that matter to patients, measures the costs over the full cycle of care, and favors a truly integrated practice. These principles have the potential to improve care while lowering overall costs to the healthcare system. Team-based models of primary healthcare and targeted integrated-practice units for investigation can improve communication among providers, and result in more coordinated and concerted care, plus earlier diagnosis. Alberta’s Thoracic Oncology Program was presented as a team-based model of investigation with an automated referral mechanism, which successfully provides faster access to diagnosis and treatment [29]. This program suggests that streamlining and integrating care will improve earlier diagnosis and patient outcomes. Value-based collaborative practices in Canada will benefit from continued efforts and support towards a multidisciplinary network among healthcare professionals where resources, data, and knowledge are transferred more efficiently.

Value-based healthcare may provide a means to overcome barriers in molecular testing, which facilitates impactful precision medicine approaches [30]. Molecular testing and comprehensive genomic profiling are used to tailor oncological treatments to the unique molecular profile of an individual’s malignancy [31]. However, inappropriate delays in testing and reporting often result in low clinical utility. Increased collaborative efforts among stakeholders was suggested towards improved access to molecular testing, and thereby to precision medicine treatment regimes.

### Calls to Action

- Increase collaboration among healthcare stakeholders by moving from simple inter- or multidisciplinary care to integrated practice units.
- Measure multidimensional patient-reported outcome and experience measures (PROMs and PREMs) in a standardized way for all patients to optimize their quality of life and trajectory of care.
- Measure costs over the entire care cycle to optimize outcomes with limited resources.

## 4. Timely Palliative Care

Palliative care has historically been stigmatized as care for end-of-life; however, by definition, palliative care represents the “active and holistic care of individuals with serious health-related suffering due to illness” [32]. The diverse symptom burden of pancreatic cancer, as well as complications due to treatment, warrant a palliative approach to disease management. The goals of palliative care include the reduction of suffering and the improvement of quality of life among patients and their caregivers [33]. Traditionally, palliative care was only delivered to patients with high symptom burden in the terminal phase of illness. However, recent palliative approaches have been integrated within oncology care to include patients earlier in the cancer continuum [32,34,35]. A Canadian retrospective cohort study has identified specialist palliative care consultation to be associated with decreased odds of experiencing aggressive care (i.e., hospitalization, emergency room visit, intensive care unit admission, etc.) at end-of-life among patients with PC [36]. Furthermore, integration of early palliative care in PC management has been correlated with a better quality of life [37] and longer overall survival [38].

Despite the ongoing efforts to integrate earlier palliative care, many patients do not access these supports due to barriers such as lack of funding, stigma of services, as well as regional and age disparities [39]. Advanced-care planning and team-based palliative care strategies may help to ensure individuals receive the care that they need, when they need it [40,41]. For example, integrated support from HCPs can help promote widespread adoption of timely palliative care. HCPs may inspire reflection on end-of-life values, encourage identification of substitute decision makers, clarify information about diagnosis and prognosis, and document discussions. Furthermore, HCPs can acknowledge the mental and emotional burden of a life-limiting diagnosis for both patients and families, and can refer to specialist mental health supports as needed. Overall, collaboration among specialist and generalist palliative care providers may help to improve patient outcomes throughout the illness trajectory.

### Calls to Action

- Make early palliative care accessible to all patients.
- Provide education and resources for patients to tailor a multidisciplinary palliative approach that is aligned with their own values.
- Implement educational and awareness initiatives on palliative care as a part of multidisciplinary patient-centered oncology.

## 5. Pancreatic Enzyme Replacement Therapy (PERT)

Pancreatic enzyme insufficiency (PEI) and diabetes—both forms of organ failure resulting from PC—can cause exocrine tissue fibrosis, ductal obstruction, decreased digestive enzyme activity, malnutrition, gland volume reduction, and decreased appetite [42]. Recent research has highlighted the importance of PERT for all patients with PC [43]. PERT is a cost-effective treatment, often prescribed to patients exhibiting PEI to normalize their digestion of fat, protein, and carbohydrates. Notably, PERT has proven advantageous by improving quality of life and survival, decreasing pancreatic and hepatic pain, and mitigating weight loss [44,45,46]. Furthermore, PERT can have a similar degree of positive impact on patient survival compared to chemotherapy [47].

Despite these benefits, PERT is not routinely administered in clinical practices for PC patients, and when it is, considerable inconsistencies exist between practices [44]. RICOCHET, a prospective observational study of patients with suspected pre-ampullary and biliary tract cancers conducted in the UK demonstrated evidence of consistent PERT prescription within organizations (be it high or low adoption of PERT) [35]; however, patients with unresectable diseases tend to be prescribed PERT at a lower frequency, despite being treated at the same organization [48]. Furthermore, the audit found no clear correlation between high or low prescribing rates in specialist care and networked referring teams. In Canada, PERT is not universally covered in all provinces, which is likely to contribute to additional discrepancies in prescription. Thus, implementing a national standardized approach for PERT may facilitate more widespread use and more precise dosing.

In support of this goal, Craig’s Cause Pancreatic Cancer Society collaborated with the Canadian Digestive Health Foundation to develop an online tool *Digest This! PERT Calculator* (https://digestthis.ca/, accessed 08/10/2024) [49]. The app enables patients to accurately calculate the recommended PERT dosage based on the user’s input food combinations, ensuring precise administration at each meal. Compared to other applications such as *PERT* Pilot [50], the *Digest This! PERT Calculator* enables a more precise recommended dosage, rather than simply tracking a patient’s trial-and-error data. By leveraging technology, patients can take control of their treatment regimen, promoting adherence and maximizing therapeutic benefits. Integration of the *Digest This! PERT Calculator* provides an avenue towards better patient outcomes and quality of life in PC.

### Calls to Action

- Educate HCPs on the influence of PEI and malabsorption on cancer-related cachexia, tolerance to treatment, quality of life, and overall survival.
- Make PERT accessible to all PC patients.
- Optimize the efficacy of PERT by widespread adoption of the PERT dosage tool.

## 6. Cancer-Reducing Treatment

Though the treatment landscape in other major cancers has improved over the past decade [51,52,53,54], treatment for PC has not benefitted from similar advances [55]. PC is characterized by a strong immune resistance, reducing its compatibility with immunotherapy treatments that are being adopted in other cancers [56,57,58,59]. Ongoing preclinical research highlights the potential of immunotherapy using a combination of natural killer T cells (NKT cells), oncolytic viruses, and checkpoint inhibitors to reducing pancreatic tumor burden [60]. The presence of NKT cells has been associated with improved outcomes in neuroblastoma [61], colorectal cancer [62], and pancreatic cancer [63,64]. Together, the presented work highlighted the early headway being made in the use of immunotherapies as treatment for PC.

Current advancements in PC surgical resection include more comprehensive pre-operative care, clarity in patient eligibility, and an improved understanding of tumor characteristics. As the role of tumor stage and metastasis in successful tumor resection becomes increasingly understood, criteria defining surgical candidacy have become more stringent and precise. Furthermore, some evidence has indicated that neoadjuvant chemotherapy may help to slow tumor growth, leading to improved surgical outcomes, particularly for patients with borderline resectable disease [65,66]. Additionally, the morbidities associated with surgery have been emphasized as key considerations during shared decision making within patient care [67].

Advances have also been made in adjuvant and neoadjuvant therapy for both resectable and borderline resectable PC. Previous clinical trials, including NEONAX [68] and SWOGS1505 [69], demonstrate that within the current treatment landscape, patients with resectable disease benefit from upfront surgery followed by adjuvant therapy. In contrast, neoadjuvant therapy has not been shown to improve outcomes for those with resectable disease, and treatment-related toxicities may delay surgical intervention. However, for patients with borderline resectable disease, there is a potential benefit of neoadjuvant treatment. A 2018 report by Versteijne et al. highlighted improved median overall survival when compared to patients who did not receive neoadjuvant therapy (18.8 months with neoadjuvant therapy vs. 14.8 months with upfront surgery) and R0 resection rates (86.8% vs. 66.9%, *p* < 0.001), but a reduction in the overall resection rates (66% vs. 81.3%, *p* < 0.001) [70]. Several clinical trials are underway, which will clarify the role of neoadjuvant therapy for PC using modern chemotherapy regimens.

Radiotherapy may also play a role in neoadjuvant treatment. The phase III PREOPANC-1 trial demonstrated improved R0 resection rates overall and disease-free survival in the group that received neoadjuvant gemcitabine and concurrent radiotherapy, when compared to the group that did not [71]. In another phase II clinical trial, a total neoadjuvant approach using FOLFIRINOX followed by individualized concurrent chemoradiotherapy demonstrated high R0 resection rates, prolonged median progression-free survival, and overall survival in patients with borderline resectable disease who went on to undergo resection [72]. However, neoadjuvant radiotherapy is not universally beneficial, as noted in the ALLIANCE A021501 phase II trial. Patients who received neoadjuvant mFOLFIRINOX and hypofractionated radiotherapy demonstrated poorer outcomes and shorter overall survival compared to those who received neoadjuvant mFOLFIRINOX alone [73]. These conflicting results suggest the need for optimized patient selection criteria, as well as chemotherapy and radiotherapy dose fractionations, to determine the most effective approach on an individualized basis.

### Calls to Action

- Alongside ongoing surgical advances, normalize discussing the potential risks and anticipated benefits of a surgical approach to further empower patient decisions.
- Continue to support research towards optimizing the role of immunotherapy in PC.
- Support clinical trials investigating neoadjuvant treatment approaches and encourage enrollment in these trials across Canada.
- Optimize selection criteria for neoadjuvant vs. adjuvant approaches alongside potential resection.

## 7. Canadian Clinical Trials

Clinical trials are a component of standard of care in oncology and are imperative in cancers with unmet needs, including PC. The goal of trials is to improve the treatment and prevention of cancer. However, there are several challenges within the current landscape of clinical trials in Canada for PC.

In Canada, many academic clinical trials are conducted through the Canadian Cancer Trials Group (CCTG), supported through philanthropy by the Canadian Cancer Society and the National Clinical Trials Network, as well as through industry and government grants. The CCTG is structured as an external network of member centers and committees, composed of a Scientific Committee, Oversight Committee, and Support Committee. The main challenges identified for clinical trials in 2023 were lack of funding, accrual, and capacity. Effective cancer trials necessitate active involvement from various stakeholders, encompassing regulatory bodies, industry partners, philanthropic organizations, researchers, and patients. Considering the role of clinical trials in expanding treatment options and improving patient outcomes [74], support for clinical trials and patient awareness of actively recruiting trials is imperative for present and future patients with PC.

### Calls to Action

- Increase financial support and other resources to enable clinical trials to be effectively conducted in Canada.
- Discuss the advantages and risks of recruiting clinical trials based on patient eligibility immediately following diagnosis.
- Educate patients on the benefits of clinical trial participation compared to standard of care—reduce the stigma.

## 8. Progress and Current Landscape

*Chemotherapy and Radiation*: While improvements in median survival for PC are limited relative to other cancers, several advancements have been observed because of chemotherapy and radiation. In the adjuvant setting for PC, median survival has progressed from observation alone (15.9 months) to 5FU (20.1 months), Gemcitabine (22.5 months), and Gemcitabine + Capecitabine (28.0 months) [75,76]. The recent PRODIGE 24/PA.6 trial—comparing FOLFIRINOX to Gemcitabine in the adjuvant setting—showcased a substantial increase in survival (54.7 months vs. 36.3 months, *p* < 0.001) [77]. Following completion of adjuvant FOLFIRINOX, adjuvant concurrent radiation can be considered in patients with an R1 resection [78]. In the metastatic setting, both FOLFIRINOX and Gemcitabine + Nab-paclitaxel have demonstrated improved survival rates [79], however, direct comparisons between the two have yet to be conducted.

The CHLOE pancreas trial (NCT04862260) is currently investigating the therapeutic potential of three cholesterol-lowering drugs (atorvastatin, ezetimibe, and Repatha) in combination with FOLFIRINOX. Primary outcome measures include safety and characterization of dose-limiting toxicities. To date, the safety cohort has been completed (n = 3), and preliminary results demonstrate feasibility, acceptability, safety, and tolerance of the trial.

In cases of resectable PC, a debate continues to persist regarding the optimal timing for chemotherapy administration (adjuvant and/or neoadjuvant), with ongoing clinical trials addressing this issue. The NeoPancOne study, for instance, is exploring the use of GATA6 expression as a predictive biomarker for perioperative chemotherapy response [80]. Additionally, the Alliance trial is actively recruiting for a randomized study of perioperative chemotherapy vs. adjuvant chemotherapy with FOLFIRINOX [81].

For locally advanced cases, clinical trials have been investigating various treatment modalities. The ABLATE study, using ablative high-dose radiation, is looking to open in Canada [82]. In this study, upfront biopsies will be performed for whole genome and transcriptome sequencing, as well as to develop patient derived organoids, to provide correlatives in addition to primary endpoints.

*Precision Medicine*: Precision medicine represents a new age for PC treatment, capitalizing on tailored drug composition based on the individual’s (or the specific tumor’s) genomic characteristics. In pancreatic ductal adenocarcinoma (PDAC), up to 40% of patients harbor an actionable mutation [83], which facilitates improved overall outcomes by way of accessing targeted therapies. Conversely, patients without these mutations, or those who do not receive targeted therapies, have poorer outcomes on average. Moreover, genomic profiling can yield insight regarding prognosis [84].

A discussion of ongoing trials focused on translating genomic data into precision therapies for PDAC, namely the EPPIC and COMPASS trials [85,86]. The goal of these studies was to investigate PC genomics, use the findings in a molecular tumor board, and find novel targeted treatments for patients, either off-label or in clinical trials.

Additionally, upcoming trials aim to leverage genomics to tailor treatments for PC patients. PASS-01 aims to evaluate the efficiency of two standard chemotherapy regimens while integrating advanced molecular profiling techniques, chemotherapy sensitivity signatures, and biomarker analysis to predict treatment response [87]. IND.243 is investigating the potential of the tumor growth inhibitor RP-6306 and gemcitabine in *KRAS* and *TP53*-mutated PC patients who have previously undergone FOLFIRINOX [88]. Similarly, RMC-6236 focuses on targeted therapy for *KRAS* G12C or *RAS* mutated PC tumors [89], identified through genetic sequencing. Collectively, these studies underscore the importance of sequencing PC tumors to guide precision therapies.

Initiatives such as Prosper-PANC (Prosper-PANC—Pancreatic Cancer Canada, n.d.) [90] and MAESTRO [91] are aiming to enable real-time genomic sequencing for Canadians, with the goal of directly impacting patient care.

*Immunotherapy*: Immunotherapies represent a promising avenue for PC, yet single agent therapeutic approaches have thus far yielded limited efficacy [92]. Similarly, combining immunotherapy with chemotherapy has shown limited success [93]. As such, the results of ongoing clinical trials will be of high interest. The DORA trial is investigating the impact of Durvalumab and oleclumab in PDAC to determine the increase in immune cell infiltration, particularly CD8+ T cells in tumor post-treatment (Durvalumab and Oleclumab in Resectable PDAC—ClinicalTrials.Gov, n.d.) [94]. Another study, IMCODE003, is assessing the efficacy of adjuvant autogene coumaran plus atezolizumab and mFOLFIRINOX vs. mFOLFIRINOX alone in resected PC [95]. These trials represent important steps in advancing treatment of PC through therapeutic approaches.

### Calls to Action

- Continue to support research in better selecting systemic therapies for patients to optimize benefits and minimize side effects.
- Determine the controlling factors in deciding the optimal time to administer chemotherapy: adjuvant, neoadjuvant, or perioperative.
- Strive toward more equitable access to clinical trials across Canada.

## 9. Conclusions

The Canadian National Pancreas Conference hosted a series of engaging discussions between healthcare providers, researchers, and students towards enriching the cross-discipline knowledgebase of current PC treatment and management. Pancreatic cancer is a multifaceted disease that warrants a multifaceted approach to treatment and management. The influence that supportive factors such as nutrition, mental health, and symptom burden have on patient quality of life and survival necessitates the fostering of a cross-disciplinary network among healthcare providers and foundational researchers, whereby experts in each respective area could be mutually inspired by the common goal of changing the outcome for patients with pancreatic cancer. With the increasing body of evidence pointing to integrated palliative care, HCPs should facilitate access to diverse palliative supports and educate patients on the advantages of them. Common goals clearly emerged from the event, including improved accessibility to genetic profiling, widespread adoption of PERT, optimized stratification between chemotherapy regimens, and continued research towards effective precision and immunotherapies. The 2023 NPC brought together HCPs to identify needs, current advances, and goals for PC research and patient care. To improve the outcomes of PC care in Canada, multidisciplinary teams should work to address the presented calls to action. The next NPC event will summarize completed initiatives and provide next steps in PC management strategies.

## Supplementary Materials

The following supporting information can be downloaded at https://www.mdpi.com/article/10.3390/curroncol31100461/s1, Supplement File S1: Target Audience Needs Assessment; Supplement File S2: 2023 National Pancreas Conference Program.

## List of Abbreviations

CAPS5	Cancer of Pancreas Screening-5
CCTG	Canadian Cancer Trials Group
CT	Computed tomography
HCP	Healthcare provider
MRI/MRCP	MRI/MR-cholangiopancreatography
NKT	Natural killer T
NPC	National Pancreas Conference
PC	Pancreatic adenocarcinoma
PDAC	Pancreatic ductal adenocarcinoma
PEI	Pancreatic enzyme insufficiency
PERT	Pancreatic enzyme replacement therapy
PROM	Patient-reported outcome measure
PREM	Patient-reported experience measure
