# Supplementary material for: Canadian National Pancreas Conference 2023: A Review of Multidisciplinary Engagement in Pancreatic Cancer Care
Source: Curr Oncol. 2024 Oct 16;31(10):6191–204. doi: 10.3390/curroncol31100461 (PMC11506161; doi:10.3390/curroncol31100461)
Supplement: Supplementary file 1 [file curroncol-3194806 National Pancreas Conference Program.pdf › curroncol-3194806 Supplemtnary Materials - peer review/curroncol-3194806 National Pancreas Conference Program.pdf]

NPC 2023

Accredited by
Canadian Association of General Surgeons (CAGS)

ANNUAL PANCREAS MEETING

NATIONAL PANCREAS CONFERENCE

November 18 - 19

Learn · Collaborate · Treat

Craig's Cause
Pancreatic Cancer Society

#NPC2023
@craigscausecan
info@craigscause.ca

Craig's Cause Pancreatic Cancer Society and The Canadian Association of General Surgeons is pleased to announce the 2023 National Pancreas Conference.

Codeveloped by

Craig's Cause
Pancreatic Cancer Society

CANADIAN ASSOCIATION OF
General Surgeons

Montréal, Québec

The National Pancreas Conference 2023 is offered as a hybrid conference, both, in-person and virtual.

TABLE OF CONTENTS

WELCOME 2

GENERAL INFORMATION 3

PROGRAM INFORMATION 5

RESEARCH GRANTS 6

AGENDA 7

SCIENTIFIC PROGRAMME COMMITTEE 8

PRESENTATIONS 9

- Keynote Speaker : Improving Patient Outcomes and Quality of Life Through Pancreatic Enzyme Replacement Therapy - PERT Calculator 10
- Healthcare (Panel): From Fragmentation and Volume to Integration and Value 11
- Diagnosis and Screening for Pancreatic Cancer (Panel) 14
- Treatment (Panel): Neoadjuvant Therapy, Immunotherapy, Ablation Therapy and Surgical Advancements 16
- Management (Panel): Palliative Care and Mental Health 19
- Clinical Trials (Panel): Advances & Current Landscapes 21

INDUSTRY SPONSORS & PARTNERS 24

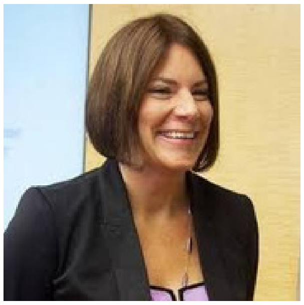

Dear Participants,

It is with great pleasure that I welcome you to Canada's Accredited National Pancreas Conference 2023. This is the only national disease-based conference dedicated to pancreatic cancer, bringing healthcare professionals together from across Canada and abroad, to learn, discuss and collaborate.

I am very happy to introduce our Health Canada approved PERT Calculator at this conference. We are confident that the PERT Calculator has the potential to make a significant impact in the field of pancreatic health, and we are excited for it to be a part of the National Pancreas Conference, where groundbreaking ideas and advancements are celebrated.

I would like to thank Dr. Walsh, for being the Chair of the National Pancreas Conference, as well as the Scientific Committee for their work on the conference. I also have to thank our many speakers, from Canada and abroad, for generously contributing to the success of this Conference through their time and expertise.

Stefanie Condon-Oldreive
Founder
Craig's Cause Pancreatic Cancer Society

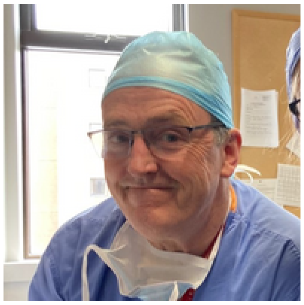

Colleagues,

I would like to welcome you the the 2023 Accredited National Pancreas Conference. This is a two-day hybrid in-person and virtual conference, November 18 & 19.
It will bring together practitioners caring for those affected by this disease, researchers investigating the basic science behind this disease as well as those looking at its treatment, and advocates for patients and families affected by pancreatic cancer. A wide variety of topics and speakers will ensure that all those attending will gain some new insights into this particularly deadly condition.

Mark Walsh, MSc, MD, FRCSC, FACS
Assistant Professor of Surgery
MOTP & HPB Surgery
Surgical Director, Liver Transplantation

Online Protocol

- Remember to post your name on your Zoom account to facilitate our attendance records, so participants can claim Section 1 MOC credits
- Keep your camera on, so our presenters can see their audience.
- Each presentation will have an interaction component for asking questions and collaborating.
- If you have a question, during the presentation, type it into our chat. Our moderators will ensure your question will be answered.
- For Technology Support, a phone number and email will be provided prior to the conference.
- Participants are encouraged to post and engage in discussions about presentations and collaborations!

Social Media #NPC2023

@CraigsCauseCan

www.linkedin.com/company/craig-s-cause-pancreatic-cancer-society

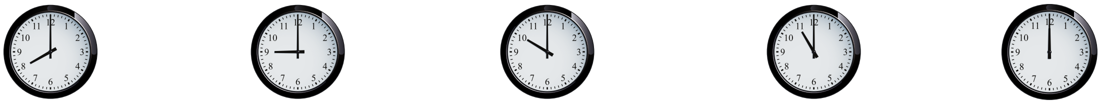

PT MT CT ET AT

NPC 2023 Venue & Host Hotel

Best Western Ville-Marie
3407 Rue Peel, Montréal
Québec H3A 1W7

Registration & Information: The Mezzanine
Conference Room. - Grand Salon
Networking - Peel Salon
Poster Presentations - Mont Royal
Social Hour - Mont Royal

Badges

NPC name badges are non transferable and to be worn and visible at all times by all delegates. They are required to access sessions and social functions.

Social Media #NPC2023

@CraigsCauseCan

www.linkedin.com/company/craig-s-cause-pancreatic-cancer-society

Learning Objectives for the National Pancreas Conference

- Describe Pancreatic Exocrine Insufficiency and the importance of considering pancreatic enzyme replacement therapy
- Discuss current advancements in treatment spanning precision medicine, immunotherapy, neoadjuvant and ablation therapy, and chemotherapy and propose their anticipated impact
- Reflect on personal practices and your province's healthcare system with respect to providing equitable and multidisciplinary treatment and management to all patients diagnosed with pancreatic cancer including palliative approaches and current clinical trials

Accreditation Statement

This event is an Accredited Group Learning Activity (Section 1) as defined by the Maintenance of Certification Program of the Royal College of Physicians and Surgeons of Canada and approved by the Canadian Association of General Surgeons. You may claim a maximum of 8 hours.

Certificate of Attendance

All delegates attending the National Pancreas Conference may receive a Certificate of Attendance. Following the conference an email will be sent with the information for you to document your credits.

Session Evaluation

We value your feedback! At the conclusion of each session participants will be asked to complete a short evaluation. At the end of the conference, once again participants will be asked to complete an evaluation on the conference delivery as a whole.

Disclosure of Conflict of Interest

The National Pancreas Conference has a formal policy regarding the need for authors and presenters to inform the National Pancreas Conference of any conflict of interest (COI). A COI includes, but is not limited to, employment, ownership of stock, membership on a standing advisory council or committee, being on the board of directors, publicly associated with a company or its products. Other potential areas of real or perceived COI could include receiving honoraria, consulting fees, or grants.

Craig's Cause Pancreatic Cancer Society is committed to these efforts by not only supporting specific pancreatic cancer research, the National Pancreas Conference but we are thrilled to reveal several research awards, at various levels of research.

Pancreatic cancer is a multifaceted disease that demands treatment and management from a variety of sectors in the healthcare field, with additional strong networks between healthcare providers and foundational researchers. Many of the unique challenges associated with pancreatic cancer precipitate from late-stage diagnosis, molecular and cellular heterogeneity, and several morbidities that largely derive from malnutrition. As reviewed by Grossberg in 2020, a multimodal approach that integrates improved screening, targeted/precision medicine approaches to treatment (including in the palliative setting), and holistic management of symptoms has proven to increase metastasis-free survival and quality of life[1].

Advancements in treatment options for pancreatic cancer have not kept pace with those for other cancers[2], but efforts continue to adapt, develop and combine approaches to effect change in the difficult-to-treat cancer.

We encourage not only multi-disciplinary approaches and teams but also multi-disciplinary approaches that combine talent from more than one province!

Winners will be awarded during conference

Poster Board Award
Award Amount: $750.00
Total of Awards: 2

The intent of the poster presentations is to encourage collaborative and interdisciplinary approaches that improve patient survival rates and quality of life.

Junior Research Grant
Award Amount: $7,500.00
Total of Awards: 2

The intent of the Junior Researcher Grant is to create opportunities that encourage collaborative and interdisciplinary approaches in the field of pancreatic cancer research. Applicants are encouraged to look at projects that will increase survival rates and improve quality of life

Faculty Research Grant
Award Amount: $75,000.00
Total of Awards: 2

The intent of a Senior Research Grant opportunity is to encourage collaborative and interdisciplinary approaches that improve patient survival rates and quality of life are key to this research grant opportunity.

AGENDA

18 Saturday

8:00-9:00 am	Welcome Breakfast
9:00-10:30 am	Keynote Speaker : Improving Patient Outcomes and Quality of Life Through Pancreatic Enzyme Replacement Therapy - PERT Calculator
10:45 - 12:00pm	Healthcare: From Fragmentation and Volume to Integration and Value
12:00 - 1:20pm	Networking Lunch
1:30 - 2:45pm	Diagnosis and Screening for Pancreatic Cancer (Panel)
3:00 - 4:15pm	Treatment (Panel): Neoadjuvant Therapy, Immunotherapy, Ablation Therapy and Surgical Advancements
4:15 - 5:30pm	BREAK
5:30-6:30pm	Meet the Speaker and Research Boards
6:30-8:30pm	Dinner and Research Awards Presentation
9:00am - 8:00pm	Research Board Presentations - available for viewing throughout the day

19 Sunday

7:30-8:15am	Breakfast
8:30 - 9:45am	Management (Panel): Palliative Care and Mental Health
10:00 -11:15am	Clinical Trials (Panel): Advances & Current Landscapes
11:30am -12:00pm	Remaining Research Awards Presentation

Craig's Cause Pancreatic Cancer Society is incredibly grateful to the volunteer members of the National Pancreas Conference 2023 Scientific Program Committee. It is with their guidance and expertise that has made this first conference of its kind available.

Chair

Dr. Mark Walsh, MD
Hepato-Pancreato-Biliary Surgeon

Committee Members

Stefanie Condon-Oldreive, Founder/Director
Craig’s Cause Pancreatic Cancer Society

Jessica Nickerson, PhD Healthcare Education Rep.,
Craig's Cause Pancreatic Cancer Society

Emmanuel Ajuwon, MD, Nova Scotia

Jeanette Boudreau, BSc, PhD, Nova Scotia

Phillip Cooper, MD, Nova Scotia

Sharlene Gill, BSc (Pharm), MD, MPH, MBA, FACP, FRCP(C),
British Columbia

Rob Grant, PhD, Ontario

Julie Hallett, MD, MSc, FRCSC, Ontario

Popi Kasvis, PhD, Québec

Diane Kelsall, MD, Ontario

Michael Moser, MD, MSc, FRCSC, Saskatchewan

Ravi Ramjeesingh, MD, PhD, Nova Scotia

Mustapha Tehfe, MD, MSc, Québec

George Zogopoulos, MD, PhD, FRCS(C), FACS, Québec

PRESENTATIONS

KEYNOTE: Improving Patient Outcomes and Quality of Life Through Pancreatic Enzyme Replacement Therapy - PERT Calculator

Dr. Keith Roberts HPB Surgeon

PRESENTATION SUMMARY:

The pancreas is critical for digestion of food and absorption of nutrients, so when pancreatic cancer is compromising the organ's function, pancreatic enzyme insufficiency can occur. Most cancers are associated with weight loss, so the additional burden posed by a reduction in digestive enzyme production amplifies this symptom for pancreatic cancer patients and the lower nutritional intake reduces their body's ability to tolerate aggressive treatment or fight the disease. Pancreatic enzyme replacement therapy replenishes the important enzymes that the pancreas is unable to produce efficiently. This session will educate participants on current best practices involving pancreatic enzyme replacement therapy and highlight its impact on improving patient outcomes. A tutorial of our new Health Canada approved PERT calculator will accompany this presentation.

LEARNING OBJECTIVES:

By the end of this session, learners will be able to:

- Describe how pancreatic malignancies lead to malnutrition and malabsorption
- Discuss the effects of PEI on patients' quality of life and survival
- Summarize the evidence supporting the use of pancreatic enzyme replacement therapy

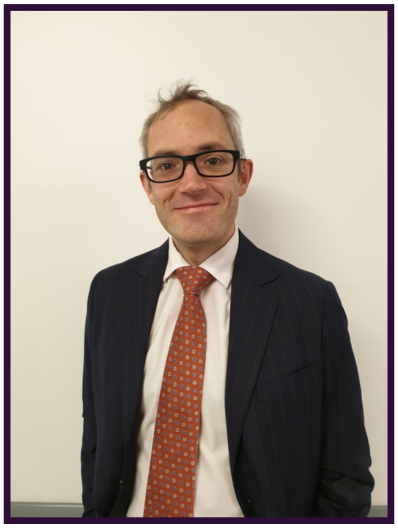

Prof Keith Roberts is a HPB and liver transplant surgeon at University Hospitals Birmingham NHS Trust and Honorary Professor at the University of Birmingham.
He is the pancreas cancer sub-speciality lead for the Royal College of Surgeons of England and Honorary Treasurer to the Pancreatic Society of Great Britain and Ireland. His research expertise is in pancreatic exocrine insufficiency, post operative pancreatic fistula and pathways to surgery for pancreatic cancer.

Healthcare: From Fragmentation and Volume to Integration and Value (Panel)

Filomena Servidio-Italiano
Colorectal Cancer Research & Action Network

Leah Stephenson
All.Can Canada

Eva Villalba, M.B.A, MSc, VBHC Green Belt
Quebec Cancer Coalition

PRESENTATION SUMMARY:

With significant stressors in the Canadian healthcare system, cancer patients are waiting extraordinarily long periods of time to be seen by a specialist, receive testing/imaging and have their treatment plans implemented. Factors such as geographical location, access to appropriate healthcare services, economical status, and culture, amongst many others, act as a barrier for patients to receive timely care. Lack of funding and a shortage of healthcare workers makes providing universal healthcare to all Canadians unattainable. It is essential that healthcare professionals collaborate with one another to coordinate care of patients and ensure patients have all of the support they need for the best outcomes. This session will educate participants on solutions to overcoming healthcare barriers and the power of collaboration to ensure patients receive the care they require in a timely fashion.

LEARNING OBJECTIVES:

By the end of this session, learners will be able to:

- Describe how the systemic efficiency of a multidisciplinary approach can reduce the burden for healthcare providers while improving the quality of life and outcomes for patients
- Propose an ideal multidisciplinary care model for optimal treatment and management of pancreatic cancer
- Comment on the degree of multidisciplinarity of care at your institution/facility—what could be improved?

Healthcare: From Fragmentation and Volume to Integration and Value (Panel)

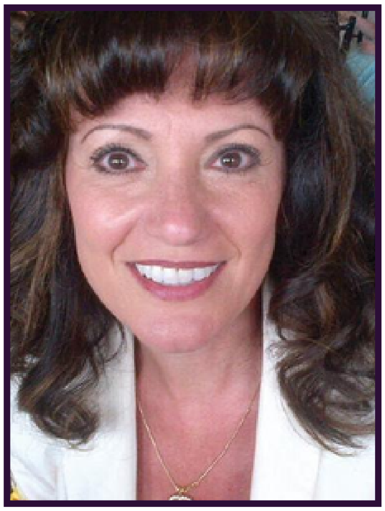

Filomena Servidio-Italiano;
Colorectal Cancer Research & Action Network

Filomena is the President and CEO of the Colorectal Cancer Resource & Action Network ("CCRAN") – a patient-focused organization championing the health and wellbeing of Canadians touched by colorectal cancer and others at risk of developing the disease. Her undergraduate and graduate work lie in the biological sciences and educational studies. As a caregiver to her father, who was afflicted with and succumbed to metastatic cancer, his journey has served as the impetus for the founding of CCRAN, one month post his passing in August of 2006. The past seventeen years have been dedicated to the cause of supporting, educating, and advocating on behalf of colorectal cancer patients and caregivers, to improve patients' quality of life and longevity through the provision of evidence-based information and access to innovative patient programs, such as CCRAN's innovative "My CRC Consultant", an online tool providing the metastatic patient with evidence-based and expert reviewed, potential therapeutic options based on the patient's tumour's molecular profile. She has been working closely with valued health care professionals across the continuum of colorectal cancer care to ensure content acumen. She is humbled to be published alongside world-renowned experts in areas such as colorectal cancer management, PROs and PROMs, early age onset colorectal cancer awareness and education, promoting national LDLT efforts, education on molecular profiling, and more.

Filomena is a strong proponent of HTA patient evidence submissions since the inception of pCODR, ensuring the patient voice is captured and well incorporated into her submissions. She prepares these submissions not only for colorectal cancer drug therapies under review, but within therapeutic areas for which there are no representative patient advocacy groups or on behalf of patient advocacy groups who may not have the capacity to make these critically important submissions. She strives to secure robust patient and caregiver participation to help identify patients' unmet needs, as well as capture their fundamentally important values, preferences, and priorities with the goal of ultimately ensuring reimbursement of effective treatments based on thoughtful and compelling input. She continues to be a passionate advocate for the Canadian cancer patient and their caregiver.

Healthcare: From Fragmentation and Volume to Integration and Value (Panel)

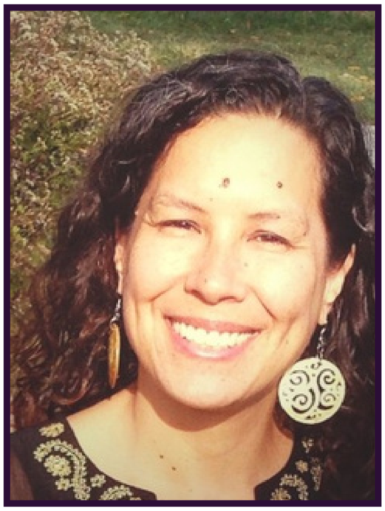

Leah Stephenson All.Can Canada

Leah Marie Migriño Stephenson has cultivated her consulting expertise to improve how we address complexity through individual transformation, organizational change and systems change. She is passionate about social change. Her experiences as a daughter and caregiver for her late mother motivate Leah. Her late mother was a physician who overcame poverty, war, gender discrimination and other systemic and personal obstacles, who struggled against meningioma and recurrent endometrial cancer, all while remaining focused on ending the suffering of others. As a strategist and lateral thinker who has developed expertise in how to better address the many intractable, complex, multi-sectoral issues we face, Leah helps identify and establish effective approaches for individual, organizational and collaborative action to help achieve needed outcomes. A wide range of frameworks and tools support this work, including but not limited to: Four Frames, Theory U, Appreciative Inquiry, Collective Impact, Liberating Structures and more.

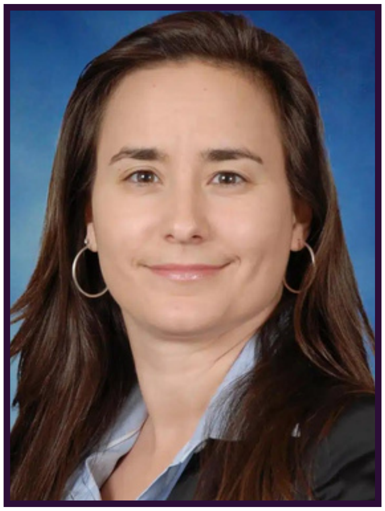

Eva Villalba, M.B.A, MSc, VBHC Green Belt; Quebec Cancer Coalition

Eva Villalba is the Executive Director of the Quebec Cancer Coalition, a non-profit patient advocacy group dedicated to improving the Quebec health-care system for people affected by cancer. Since 2008, she has been involved in health-care charities, advocacy groups and non-profits. She graduated from McGill University with a double major in Psychology and Applied Linguistics, then completed an MBA from HEC Montréal with the objective to apply best business practices to the non-profit sector. Eva is passionate about good governance, public policy, community impact, health-care reform and social innovation. She is a volunteer board member of the Artificial Intelligence Impact Alliance (AIIA) and sees great potential in applying AI and technological innovations to the health-care sector to prevent and improve health outcomes.

Eva is the first Quebecker and Canadian to obtain a graduate degree in Value-Based Health Care (VBHC) with an MSc. in Health Care Transformation at the University of Texas at Austin with Professor Elizabeth Teisberg and other VBHC experts. Eva is a fierce patient advocate and believes strongly in the potential of the Quebec health-care system to be a world-class leader in Value-Based Care. Eva has an extensive international network of Value-Based Health Transformation Leaders, has a VBHC Green Belt Certification from VBHC Center Europe and is their official Canadian Ambassador. She is also the co-chair of the Resilient Healthcare Coalition (RHC), a diverse group of health system leaders across the public, private, and non-profit sectors committed to helping Canadian health care systems enhance patient care and improve health outcomes by becoming faster, nimbler, and better able to adapt to future shocks.

Diagnosis and Screening for Pancreatic Cancer (Panel)

Dr. George Zogopoulos
McGill University Health Centre

Dr. Masoom Haider
Mount Sinai Hospital

PRESENTATION SUMMARY:
Pancreatic cancer symptoms are often overlooked by patients or are not noticed until the later stages of the disease. With minimal treatment options when diagnosed in later stages, it is imperative that the public is aware of symptoms and screening methods for pancreatic cancer. According to the Canadian Cancer Society, an estimated 6,900 Canadians were diagnosed with pancreatic cancer in 2022, and a whopping 5,700 of those diagnosed died. Risk factors such as, family history of pancreatic cancer and inherited genetic mutations, amongst other factors, make screening imperative for early diagnosis. This session will educate participants on risk assessment, inheritable gene mutations and biomarker testing to assist in targeted treatment choices, diagnosis and detection.

LEARNING OBJECTIVES:
By the end of this session, learners will be able to:

- Describe the current limitations with screening for pancreatic cancer
- Summarize recent and/or ongoing research that is contributing to improving upon current screening strategies
- Discuss how inefficient screening influences patient outcomes

Diagnosis and Screening for Pancreatic Cancer (Panel)

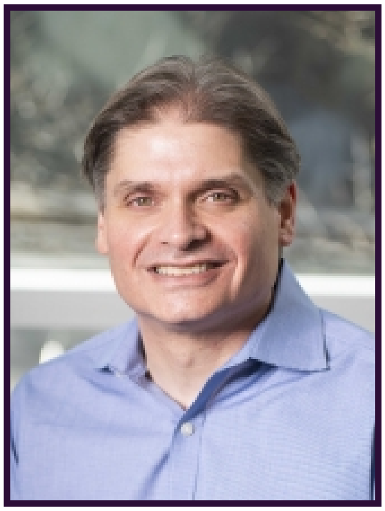

Dr. George Zogopoulos, McGill University Health Centre

George Zogopoulos, MD (Toronto), PhD (McGill) is an Associate Professor of Surgery and Oncology at McGill University, a staff surgeon at the McGill University Health Centre (MUHC), and a scientist at the Research Institute of the MUHC and the Rosalind and Morris Goodman Cancer Institute. He is a senior clinical research scholar of the Fonds de recherche du Québec - Santé and recipient of the Michal & Renata Hornstein Career Award for Surgical Excellence, with a translational research program studying the genetics and oncogenomics of pancreaticobiliary cancers.
His clinical practice focuses on the surgical treatment of pancreatic, biliary and hepatic malignancies as well as abdominal organ transplantation with an interest in transplant oncology.

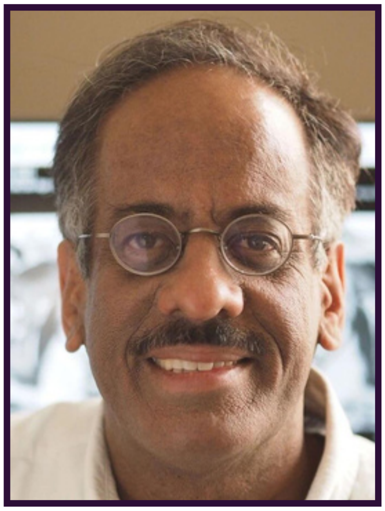

Dr. Masoom Haider, Mount Sinai Hospital

Dr. Masoom Haider is a radiologist and clinician scientist at the University of Toronto in the Joint Department of Medical Imaging. His research focuses on image biomarker validation using multiparametric MRI and CT technologies. His team uses machine learning and artificial intelligence methods combined with imaging biomarkers to develop predictive and prognostic radiomics signatures for pelvic cancers, including prostate, pancreas, kidney and liver cancers.
Dr. Haider is the imaging lead on national MRI trials in prostate cancer and has worked on guideline development for the use of multiparametric prostate MRI (mpMRI) for prostate cancer, namely the Pi-Rads standard. He holds a Chair in Artificial Intelligence, Imaging Biomarkers and Radiomics at the Lunenfeld-Tanenbaum Research Institute, Sinai Health System. In the Joint Deptartment of Medical Imaging and Lunenfeld-Tanenbaum Research Institute he leads the AI, Radiomics and Oncologic Imaging Research Lab. He collaborates with oncologists, computer scientists, engineers, radiologists and biomedical physicists.
Dr. Haider has held peer-reviewed grants from Prostate Cancer Canada, the Canadian Cancer Society Research Institute, Cancer Care Ontario and the Ontario Institute for Cancer Research for MRI-related prostate and pancreatic cancer research.

Treatment: Neoadjuvant Therapy, Immunotherapy, Ablation Therapy and Surgical Advancements (Panel)

Dr. Brent Johnston
Beatrice Hunter Cancer Research Institute

Dr. Christina Kim
CancerCare Manitoba

Dr. Boris Gala-Lopez
QEII Health Science Centre,
Dalhousie University

Dr. Sylvia Ng
Sunnybrook
Health Sciences Centre

PRESENTATION SUMMARY:

Currently, the only potentially curative treatment for pancreatic cancer is surgery. However, pancreatic tumours are only considered operable/resectable when the malignancy is detected in its early stages, before metastasis. Given the vague and late-presenting symptoms of pancreatic cancer, early diagnosis is rare, reducing the accessibility to curative treatment. Neoadjuvant therapy approaches aim to decrease tumour burden and grant more patients the option to undergo potentially-curative surgery. Ablation therapy employs various physical treatments such as radiofrequency, microwaves, ethanol, and freezing (cryosurgery) to destroy tumor cells. These treatments, along with surgical advancements, increase the likelihood of curing a patient's pancreatic cancer. This session will educate participants on current practices in neoadjuvant therapy and ablation therapy, as well as ongoing research and innovation, which aims to increase access to curative surgical treatment and improve outcomes for later-diagnosed patients.

LEARNING OBJECTIVES:

By the end of this session, learners will be able to:

- Describe why surgery is inaccessible to the majority of pancreatic cancer patients and discuss current advancements enabling surgical resection of borderline-resectable malignancies
- Review the benefits and current landscape of neoadjuvant approaches
- Describe the challenges with using conventional ablation therapy approaches in pancreatic cancer and discuss current strategies that are making it more amenable to pancreatic cancer

Treatment: Neoadjuvant Therapy, Immunotherapy, Ablation Therapy and Surgical Advancements (Panel)

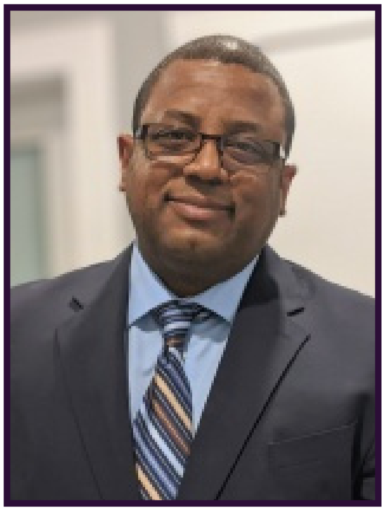

Dr. Boris Gala-LopezQEII Health Science Centre, Dalhousie University

Dr. Boris Gala-Lopez is an associate professor of surgery, microbiology, immunology, hepatobiliary and multi–organ transplant surgeon at Dalhousie University. He received his medical degree from Havana Institute of Medical Sciences, Cuba, where he continued his medical residency. Further, he obtained his master's in medical informatics from Erasmus University of Rotterdam in Netherlands, followed by a PhD in Experimental Surgery from University of Alberta.
He completed his postdoctoral training in Multi-Organ Transplant Surgery at University of Alberta Hospital before joining Dalhousie University. Dr. Gala-Lopez's research interests focus on developing new strategies to optimize organ and tissue preservation for transplantation

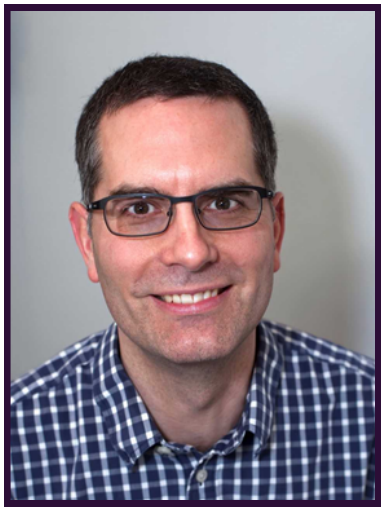

Dr. Brent Johnston
Beatrice Hunter Cancer Research Institute

Dr. Brent Johnston is Professor and Head of the Department of Microbiology & Immunology at Dalhousie University and a Senior Scientist with the Beatrice Hunter Cancer Research Institute. He obtained his PhD from the University of Calgary and completed a postdoctoral fellowship at Stanford University. Dr. Johnston's research program uses models of cancer and autoimmune disease to understand the role of natural killer T (NKT) cells in regulating immune responses and disease outcomes. Dr. Johnston's group is currently working to develop cancer therapies that incorporate NKT cell-based immunotherapy in combination with select chemotherapeutics, oncolytic viruses, and immune checkpoint antibodies.

Treatment: Neoadjuvant Therapy, Immunotherapy, Ablation Therapy and Surgical Advancements (Panel)

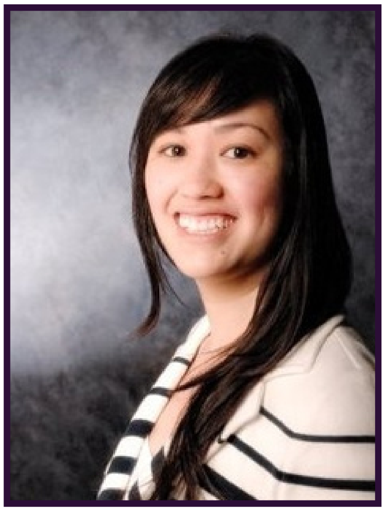

Dr. Christina Kim, CancerCare Manitoba

Dr. Christina Ai-Kheng Kim is an assistant professor of internal medicine at the Max Rady College of Medicine at the University of Manitoba. Dr. Kim serves as the research director for the medical oncology residency program committee. She is also a medical oncologist at CancerCare Manitoba. Dr. Kim was awarded a Canadian Institute of Health Research (CIHR) and Canadian Association of Medical Oncologists (CAMO)-funded fellowship focused on symptom burden and prognostic and predictive markers in pancreatic cancer.

Dr. Kim is an active member of the gastrointestinal and breast disease site groups at CancerCare Manitoba.

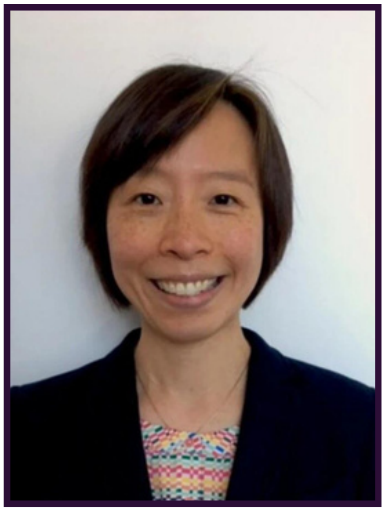

Dr. Sylvia Ng
Sunnybrook Health Sciences Centre

Dr. Sylvia Ng completed her medical training at the University of British Columbia, radiation oncology residency training and PhD in Medical Biophysics at the University of Toronto, and fellowship at the University of Texas MD Anderson Cancer Centre. Her clinical interests include pancreatic and hepatobiliary cancers, gastrointestinal cancers, SBRT, and MR-guided radiotherapy. She is the pancreatic cancer MR-LINAC lead at the Sunnybrook Odette Cancer Centre. Her scientific expertise lies in pancreatic cancer, tumor microenvironment, and molecular pharmacology. Dr. Ng has published in the areas of translational research, cancer biology, targeted therapy, drug development, and biomarkers in addition to clinical radiation oncology.

Management: Palliative Care and Mental Health (Panel)

Valerie Cooper, NP
South East Local Health Integration Network

Dr. Breffni Hannon
Princess Margaret Cancer Centre

PRESENTATION SUMMARY:
Often times, palliative care is associated with end of life care when in fact, palliative care is supportive care and should be used in tandem with other treatments. Patients who receive palliative care have a higher quality of life and live longer. Part of a palliative care approach should include mental health supports. Addressing mental health concers is crucial in helping patient's process feelings, thoughts and navigate their diagnosis. Mental health care can include counseling, coping skills, medication and other techniques to help patients who are struggling with depression, anxiety or grief as a result of their diagnosis. In this session, participants will learn about the importance of palliative care, what supports are available for patients and how to help patients get access to the mental health care they need.

LEARNING OBJECTIVES:
By the end of this session, learners will be able to:
- Discuss the role of palliative care in determining a patient's outcome
- Describe the roles of various disciplines within the healthcare field that contribute to palliative care and overall disease management
- Discuss how mental health factors may influence a patient's quality of life/outcome and describe the resources that should be made available to a patient and their family across a pancreatic cancer journey

Management: Palliative Care and Mental Health (Panel)

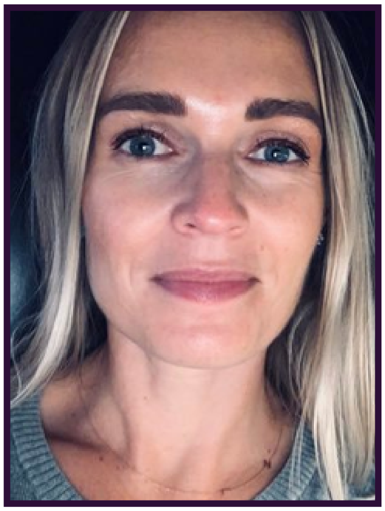

Valerie Cooper, NP
South East Local Health Integration Network

Valerie Cooper is a Hospice Palliative Care Nurse Practitioner with Home and Community Care Support Services South East. She sees patients in their homes in a variety of geographies, from urban to remote. She works in a shared-care model with patients' existing primary care providers to support patients with malignancies and end-stage organ diseases to die in their preferred place of death. She lectures in the undergraduate program at Queen's School of Nursing and has developed and taught an elective undergraduate course on hospice palliative care nursing. She has also contributed to a continuing education course for nurse practitioners on palliative care through the University of Toronto. Valerie is a member of the Dying with Dignity Canada's Clinician Advisory Council, the Canadian Association of MAiD Assessors and Providers (CAMAP) Provincial and Territorial Advisory Council, as well as the CAMAP National MAiD Curriculum Working Group. She is the chair of her local MAiD Community of Practice, as well as a LEAP facilitator for Pallium Canada. She has spoke across Canada about both palliative care and MAiD

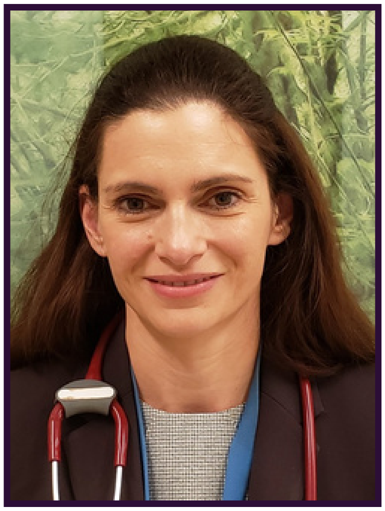

Dr. Breffni Hannon
Princess Margaret Cancer Centre

Dr. Breffni Hannon leads the palliative care program at the Princess Margaret Cancer Centre in Toronto. Originally from Ireland, she moved to Canada for a 2-year clinical-research fellowship in palliative care in 2011 and never left! She is a clinician-investigator whose research interests include developing models of early palliative care for patients with advanced cancer; exploring the needs of caregivers; and promoting advance care planning..

Clinical Trials: Advances & Current Landscapes (Panel)

Dr. Anne Gangloff
Associate Professor Laval University

Dr. Sharlene Gill
BC Cancer

Dr. Ravi Ramjeesingh
QEII Health Science Centre, Dalhousie University

Dr. Erica Tsang
Princess Margaret Cancer Centre

PRESENTATION SUMMARY:

Evidence shows that patients who participate in clinical trials have better outcomes and improved quality of life. The ever evolving research and innovation within the medical world provides hope and momentum to eventually find a curative treatment for pancreatic cancer when surgery is not an option. This session will educate participants on the current landscapes and new advancements in clinical trials, including up and coming clinical trials and current clinical trials that are showing promise.

LEARNING OBJECTIVES:

By the end of this session, learners will be able to:

- Describe the role of clinical trials in changing the statistics associated with pancreatic cancer and discuss the challenges associated with nihilism-driven low-enrollment
- Discuss the findings and impact that past clinical trials have had on changing the standard of care for pancreatic cancer
- Propose a novel clinical trial based on what you have learned in other NPC sessions

Clinical Trials: Advances & Current Landscapes (Panel)

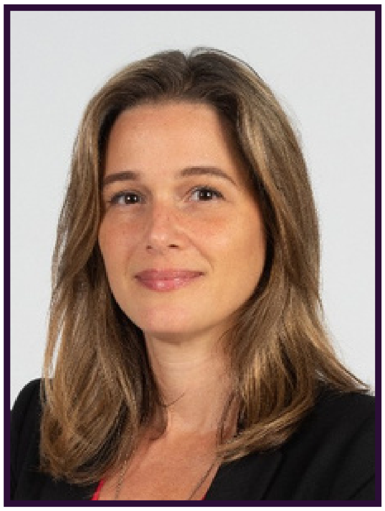

Dr. Anne Gangloff
Associate Professor Laval University

Dr. Anne Gangloff is a Clinical University Researcher and Assistant Professor at Laval University. She is also a Clinical Researcher at FRQS, Regular Researcher at the Cancer Research Centre at Laval U and Associate Researcher in the Endocrinology and Nephrology axis of the CHU de Québec-Université Laval Research Center. Dr. Gangloff is a physician specializing in laboratory medicine, including clinical pathology and medical biochemistry and is a doctor at the CHUL Lipid Diseases Clinic. She treats both pediatric and adult dyslipidemic patients and also treats patients with cardiovascular diseases. Dr. Gangloff's research interests include cholesterol and its role in cancer progression.

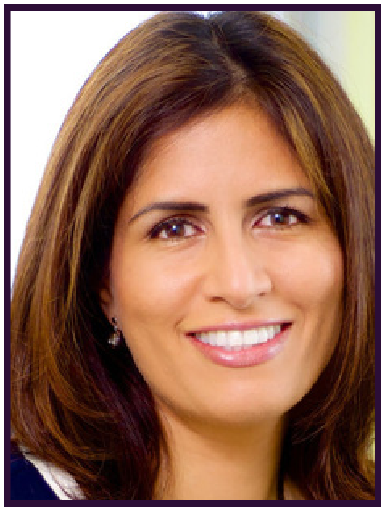

Dr. Sharlene Gill
BC Cancer

Dr. Sharlene Gill is a medical oncologist specializing in gastrointestinal (GI) malignancies at BC Cancer – Vancouver. She received a BSc. in Pharmacy, and MD from the University of British Columbia in 1996 followed by residencies in Internal Medicine and Medical Oncology, subsequently completing a fellowship in Gl Oncology at the Mayo Clinic (Rochester, MN) and a Masters of Public Health from the Harvard School of Public Health (Boston, MA) before returning to Vancouver where she is presently Professor of Medicine at the University of British Columbia. In 2017, she completed an MBA from the Kenan-Flagler School of Business at the University of North Carolina. Her areas of clinical expertise are in colorectal, pancreatic and hepatobiliary cancers. She is actively engaged in education and clinical research, with over 100 peer-reviewed publications and book chapters to her credit.

Clinical Trials: Advances & Current Landscapes (Panel)

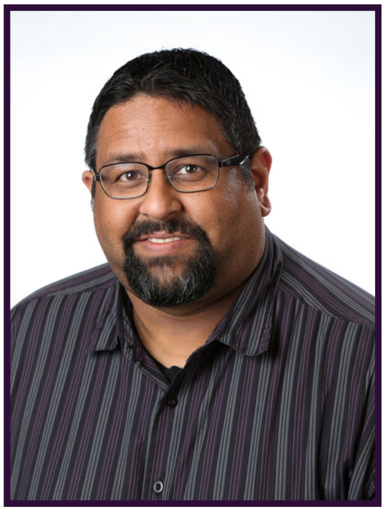

Dr. Ravi Ramjeesingh
QEII Health Science Centre, Dalhousie University

Dr. Ramjeesingh holds an MD and PhD from the University of Toronto and completed residency training in internal medicine and medical oncology through Queen's University. He then undertook a two-year clinical trial methodology and translational research fellowship with the NCIC Clinical Trials Group at Queen's.

His work focused on the appropriate timing of administration of adjuvant chemotherapy to patients with breast cancer.

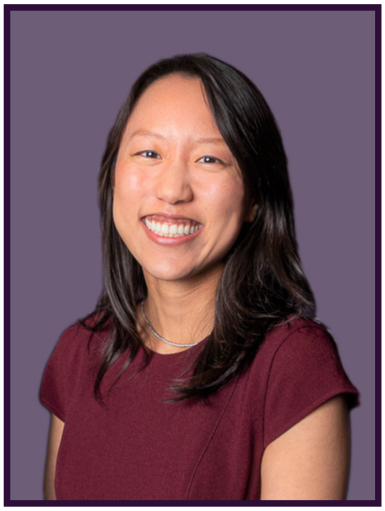

Dr. Erica Tsang
Princess Margaret Cancer Centre

Dr. Erica Tsang is a GI medical oncologist at the Princess Margaret Cancer Centre and an Assistant Professor at the University of Toronto. She completed her Internal Medicine and Medical Oncology training at the University of British Columbia, followed by further training in GI and early phase oncology at the University of California San Francisco.

Dr. Tsang also completed a Masters of Public Health at Harvard University. Her research interests involve genomics and clinical trials, with a focus on pancreatic cancer.

The National Pancreas Conference would like to thank the following sponsors and partners. With your help, we are working towards a world where surviving pancreatic cancer is expected.

Craig's Cause Pancreatic Cancer Society
P.O. Box 8561
Halifax, N.S. B3K 5M3
1.877.212.9582
www.craigscause.ca
info@craigscause.ca

#CHANGETHEOUTCOME
