# Supplementary material for: Canadian National Pancreas Conference 2023: A Review of Multidisciplinary Engagement in Pancreatic Cancer Care
Source: Curr Oncol. 2024 Oct 16;31(10):6191–204. doi: 10.3390/curroncol31100461 (PMC11506161; doi:10.3390/curroncol31100461)
Supplement: Supplementary file 1 [file curroncol-3194806 Target Audience Needs Assessment.pdf › curroncol-3194806 Supplemtnary Materials - peer review/curroncol-3194806 Target Audience Needs Assessment.pdf]

Needs Assessment

1. Literature Review

Pancreatic cancer is a common malignancy of the gastrointestinal system. Most patients are diagnosed in late stages, with a five-year survival rate of just 5%. Pancreatic cancer is projected to be the second leading cause of cancer-related death in the next two years[1], with significant disparities in treatment accessibility and outcomes based on geography, race and ethnicity[2–7]. A major objective of the National Pancreas Conference is to shed light on the systemic biases that result in disparities in the treatment efficacy and outcomes of racially visible patients. A review by Noel and Fiscella discusses that black and Hispanic patients are more often treated at low volume institutions and are less likely to undergo surgery, which is reflected by higher mortality rates[4]. We hope that providing a space for open conversation around these biases will inspire thorough inspection of each participant's practice to identify small changes they can make to reverse these statistics.

The stage of diagnosis is a major prognostic factor for patients with pancreatic cancer, and due to the disease's vague and late-presenting symptoms, it is most often diagnosed at late stages. It has also been missed in medical images, as underscored by Kang et al. in two recent studies[8,9]. As reviewed by Satyanada et al. in 2019, 80-85% of pancreatic cancer patients are diagnosed with locally advanced or metastatic disease, leaving only 15-20% of patients eligible for surgical resection—the only potentially curative treatment[1]. A review by Strobel et al. highlights the work being done to make more patients eligible for surgery (e.g. by providing neoadjuvant therapy prior to resection) and to improve surgical outcomes[10]. Benefits of immunotherapy as pre-conditioning and in combination with surgery may further improve outcomes for patients with pancreatic cancer. While immunotherapy for pancreatic cancer has been impeded by the low permeability of stroma surrounding a PDAC tumor, current research is showing promise towards the deployment of immunotherapy strategies in pancreatic cancer[11–14]. Additionally, efforts are being made to facilitate earlier diagnosis and genetics-informed precision medicine[15–17], which are expected to mobilize more treatment options and better outlook for pancreatic cancer patients[18–20]. Artificial intelligence and machine learning-based approaches are being highlighted in a dedicated session within the conference to underscore the ongoing innovations to improve our ability to detect tumors when they are still in the early stages[21,22].

Given the challenges associated with late diagnosis, many patients are not candidates to any treatment as they are too deconditioned or malnourished. Low rates of consultation with an oncologist have been discussed[23,24], which elicits the high frequency of dismissing palliative care. Although many patients are diagnosed at advanced stages, there are benefits to pursuing treatment other than the hope of being cured. The healthcare community is not just treating tumors, they're treating people—which demands the consideration of each individual's values and goals in the management of their disease. The idea of treating a patient with the objective of maximizing their quality of life is an important one that seems too often overlooked. This is where holistic approaches have to step in and consider the whole person. As reviewed by Yang in 2020, this requires a paradigm shift from a "Surgery First" outlook to one that integrates a multi-disciplinary team and an open discussion on the goals of care[25]. It's a big ask, that demands strong communication between sectors of the healthcare system, but providing patient-centered supportive care will make or break the rest of that patient's experiences. Recently, Lees et al. demonstrated that consultation with palliative care led to better care at the end of life[26]. Promising research ongoing, with objectives of facilitating earlier diagnosis. The average pancreatic cancer patient lives for one year following diagnosis, but with the right treatment plan, patients can live longer but also with a better quality of life than patients receiving only supportive therapy[27,28]. With a personalized approach, a patient's quality of life can be

drastically improved, but the complexity of this disease relies on collaboration between various sectors of the healthcare system. **The objective of the National Pancreas Conference is to bring together experts within the healthcare community (e.g. medical oncologists, HPB surgeons, nurse practitioners, pharmacists, etc.) as well as foundational researchers to remind them of their common goals of improving the lives of people living with pancreatic cancer, and provide and setting for communication about ongoing challenges and treatment opportunities in the research pipeline.** This conference will provide a platform for these experts to share their knowledge with each other and with impressionable residents (students) who are training in the fields of medical oncology, general and HPB surgery, oncology pharmacy, nursing, etc. We aim to foster a cohesive, interdisciplinary group whose combined expertise will facilitate improved detection, treatment and quality of life for patients with pancreatic cancer.

Advancements in treatment options for pancreatic cancer have not kept pace with those for other cancers[29], but efforts continue to adapt, develop and combine approaches to effect change in the difficult-to-treat cancer. For this reason, it is critical that a patient's healthcare team is informed on the clinical trials that may be accessible to the patient. The World Pancreatic Cancer Coalition has reported that patients who participate in clinical trials have better outcomes and quality of life than those who do not[30]. Eligibility criteria for clinical trials vary with many requiring that patients have not received prior treatment, so it is critical that these options are made available to the patient and thoroughly explored very early after diagnosis. It is our hope that the session on clinical trials within our conference will underscore the viability of these up-and-coming treatments to improve the prognoses of pancreatic cancer patients, and invite healthcare providers to recommend their patients participate in clinical trials when they have a reasonable chance of benefitting from them.

Precision medicine and immunotherapy are improving the outcomes and quality of life for patients with an array of cancers. Precision medicine and immunotherapy have the potential to improve the survival of patients with pancreatic cancer. However, progresses have been less significant than for other malignancies[31–39]. In the absence of precision medicine, the patient who has a high abundance of the drug targets for the standard chemotherapy will respond more positively while the treatment will not be as effective for patients whose tumors exhibit different profiles. The Know Your Tumor programme in the U.S. provides multi-omic analysis that can help in tailoring treatment based on the actionable molecular profile of each patient's tumor. It was shown in a 2020 report by Pishvaian et al. that matching the treatment to the actionable alterations improved the two-year survival rate by approximately 30%[40]. Given the promise of these targeted treatment approaches, we have dedicated a session of the conference to highlighting the important advancements being made with the objective that they will become increasingly accessible and effective in the coming years.

With increasing resolutions of analysis and research, it is now understood that pancreatic cancer is not a uniform disease at the cellular and molecular levels. This means that the genetic, but also the structural and immunologic landscape of a tumor varies even within the same patient. Perhaps uniquely, the immune system is able to recognize and react to a variety of tumor cell phenotypes. Although checkpoint-based immunotherapy has been largely ineffective against pancreatic cancer, new cell-based and combination immunotherapies are showing promise in pancreatic cancer[41–43]. Alone, and in combination with other approaches, strategies to maximize immune cell reactivity hold high potential for treatment of pancreatic cancer. Our session on immunology and immunotherapy in pancreatic cancer will explore these cutting-edge approaches and discuss opportunities for immunotherapy in patients with pancreatic cancer.

Wholistic and supportive care relies on a multi-disciplinary team of professionals, which even extends beyond the scope of the healthcare community. A common symptom of pancreatic cancer is weight loss, and as the disease progresses, cachexia can accelerate the loss of muscle mass[44]. Given the frequent and rapid weight loss associated with pancreatic cancer as well as the fact that the pancreas is integral in digestion and is of course compromised by the disease, the role of optimal nutrition in the management of

pancreatic cancer can not be overstated, including the incorporation of supplemental enzymes[45–52]. A 2018 multivariate analysis by De La Iglesia-Garcia et al. showed that two factors influenced a patient's survival following a metastatic pancreatic cancer diagnosis—1) receipt of PERT and 2) chemotherapy[53]. Therefore, we have dedicated the keynote session of this year's National Pancreas Conference to these important topics. It is our aim to highlight the positive influence that dietary considerations can have on improving the patient's quality of life, so various members of each patient's healthcare team will make time for these discussions and consider referring patients to a dietician to offer this critical type of supportive care.

Overall, the National Pancreas Conference is aiming to bridge some of the still existing gaps between professionals and researchers who are working to improve the lives of patients with pancreatic cancer. With many studies pointing to improved outcomes when patients are treated by multi-disciplinary teams working together with foundational researchers, we hope to inspire a more holistic approach for the management of this complex disease, including a widespread adoption of pancreatic enzyme replacement therapy. By bringing together this diverse set of expertise, we hope to encourage interdisciplinary connections that are indispensable in strengthening the collective force needed to fight pancreatic cancer.

References

1. Satyananda, V., Gupta, R., Hari, D. M., Yeh, J. & Chen, K. T. Advances in translational research and clinical care in pancreatic cancer: Where are we headed? *Gastroenterol. Res. Pract.* **2019**, (2019).

2. Tavakkoli, A. *et al.* Racial Disparities and Trends in Pancreatic Cancer Incidence and Mortality in the United States. *Clin. Gastroenterol. Hepatol.* **18**, 171-178.e10 (2020).

3. Nipp, R. *et al.* Disparities in cancer outcomes across age, sex, and race/ethnicity among patients with pancreatic cancer. *Cancer Med.* **7**, 525–535 (2018).

4. Noel, M. & Fiscella, K. Disparities in Pancreatic Cancer Treatment and Outcomes. *Heal. Equity* **3**, 532–540 (2019).

5. Shah, A. *et al.* Trends in Racial Disparities in Pancreatic Cancer Surgery. *J. Gastrointest. Surg.* **17**, 1897–1906 (2013).

6. Singal, V., Singal, A. K. & Kuo, Y. F. Racial disparities in treatment for pancreatic cancer and impact on survival: A population-based analysis. *J. Cancer Res. Clin. Oncol.* **138**, 715–722 (2012).

7. EK, Y. *et al.* Impact of Geography on Care Delivery and Survival for Noncurable Pancreatic Adenocarcinoma: A Population-Based Analysis. *J. Natl. Compr. Canc. Netw.* **18**, 1642–1650 (2020).

8. J, K. *et al.* The implications of missed or misinterpreted cases of pancreatic ductal adenocarcinoma on imaging: a multi-centered population-based study. *Eur. Radiol.* **31**, 212–221 (2021).

9. JD, K., SE, C. & AF, C. Factors associated with missed and misinterpreted cases of pancreatic ductal adenocarcinoma. *Eur. Radiol.* **31**, 2422–2432 (2021).

10. Strobel, O., Neoptolemos, J., Jäger, D. & Büchler, M. W. Optimizing the outcomes of pancreatic cancer surgery. *Nat. Rev. Clin. Oncol.* **16**, 11–26 (2019).

11. Leidner, R. *et al.* Neoantigen T-Cell Receptor Gene Therapy in Pancreatic Cancer. *N. Engl. J. Med.* **386**, 2112–2119 (2022).

12. Liu, L. *et al.* Nanocarriers for pancreatic cancer imaging, treatments, and immunotherapies. *Theranostics* **2022**, 1030–1060 (2022).

13. Ullman, N. A., Burchard, P. R., Dunne, R. F. & Linehan, D. C. Immunologic Strategies in Pancreatic Cancer: Making *Cold* Tumors *Hot*. *J. Clin. Oncol.* **40**, 2789–2805 (2022).

14. Yeo, D., Giardina, C., Saxena, P. & Rasko, J. E. J. The next wave of cellular immunotherapies in pancreatic cancer. *Mol. Ther. - Oncolytics* **24**, 561–576 (2022).

15. Connor, A. A. & Gallinger, S. Pancreatic cancer evolution and heterogeneity: integrating omics and clinical data. doi:10.1038/s41568-021-00418-1.

16. Ferguson, S. *et al.* Single-EV analysis (sEVA) of mutated proteins allows detection of stage 1 pancreatic cancer. *Sci. Adv.* **8**, 3453 (2022).

17. Zhu, Z. *et al.* An Enzyme-Activatable Aggregation-Induced-Emission Probe: Intraoperative Pathological Fluorescent Diagnosis of Pancreatic Cancer via Specific Cathepsin E. *Adv. Mater.* **34**, (2022).

18. Gorris, M., Hoogenboom, S. A., Wallace, M. B. & van Hooft, J. E. Artificial intelligence for the management of pancreatic diseases. *Dig. Endosc.* 231–241 (2020) doi:10.1111/den.13875.

19. Kenner, B. *et al.* Artificial Intelligence and Early Detection of Pancreatic Cancer: 2020 Summative Review. *Pancreas* **50**, 251–279 (2021).

20. Sinkala, M., Mulder, N. & Martin, D. Machine Learning and Network Analyses Reveal Disease Subtypes of Pancreatic Cancer and their Molecular Characteristics. *Sci. Rep.* **10**, 1–14 (2020).

21. Qiao, Z., Ge, J., He, W., Xu, X. & He, J. Tomography Image Features Combined with Serum Tumor Markers for Diagnosis of Pancreatic Cancer. (2022) doi:10.1155/2022/8979404.

22. Mukherjee, S. *et al.* Radiomics-Based Machine-Learning Models Can Detect Pancreatic Cancer on Prediagnostic CTs at a Substantial Lead Time Prior to Clinical Diagnosis. (2022) doi:10.1053/j.gastro.2022.06.066.

23. MN, M. *et al.* Low rates of specialized cancer consultation and cancer-directed therapy for noncurable pancreatic adenocarcinoma: a population-based analysis. *CMAJ* **191**, E574–E58 (2019).

24. Mavros, M. N. *et al.* Low rates of specialized cancer consultation and cancer-directed therapy for noncurable pancreatic adenocarcinoma: a population-based analysis. *CMAJ* **191**, E574–E580 (2019).

25. Yang, Y. Current status and future prospect of surgical treatment for pancreatic cancer. *Hepatobiliary Surg. Nutr.* **9**, 89–91 (2020).

26. C, L., S, W., N, L., T, Y. & R, R. Palliative care consultation and aggressive care at end of life in unresectable pancreatic cancer. *Curr. Oncol.* **26**, 28–36 (2019).

27. Deng, Y. *et al.* Determinants and prognostic value of quality of life in patients with pancreatic ductal adenocarcinoma. *Eur. J. Cancer* **92**, 20–32 (2018).

28. Ferrell, B. R. *et al.* Integration of palliative care into standard oncology care: American society of clinical oncology clinical practice guideline update. *J. Clin. Oncol.* **35**, 96–112 (2017).

29. Doleh, Y., Lal, L. S., Blauer-Petersen, C., Antico, G. & Pishvaian, M. Treatment patterns and outcomes in pancreatic cancer: Retrospective claims analysis. *Cancer Med.* **9**, 3463–3476 (2020).

30. Tempero, M. A. *et al.* Pancreatic adenocarcinoma: Clinical practice guidelines in oncology. *JNCCN J. Natl. Compr. Cancer Netw.* **8**, 972–1017 (2010).

31. Leinwand, J. & Miller, G. Regulation and modulation of antitumor immunity in pancreatic cancer. *Nat. Immunol.* **21**, 1152–1159 (2020).

32. Regel, I., Mayerle, J. & Mukund Mahajan, U. Current strategies and future perspectives for precision medicine in pancreatic cancer. *Cancers (Basel).* **12**, (2020).

33. Takeda, Y. *et al.* Immuno-Surgical Management of Pancreatic Cancer with Analysis of Cancer Exosomes. *Cells* **9**, 1–21 (2020).

34. Schizas, D. *et al.* Immunotherapy for pancreatic cancer: A 2020 update. *Cancer Treat. Rev.* **86**, (2020).

35. Wang, S. *et al.* Tumor microenvironment in chemoresistance, metastasis and immunotherapy of pancreatic cancer. *Am. J. Cancer Res.* **10**, 1937–1953 (2020).

36. Bazzichetto, C. *et al.* Morphologic and molecular landscape of pancreatic cancer variants as the basis of new therapeutic strategies for precision oncology. *Int. J. Mol. Sci.* **21**, 1–25 (2020).

37. Froeling, F., Casolino, R., Pea, A., Biankin, A. & Chang, D. Molecular Subtyping and Precision Medicine for Pancreatic Cancer. *J. Clin. Med.* **10**, 149 (2021).

38. Torres, C. & Grippo, P. J. Pancreatic cancer subtypes: a roadmap for precision medicine. *Ann. Med.* **50**, 277–287 (2018).

39. Aguirre, A. J. *et al.* Real-time genomic characterization of advanced pancreatic cancer to enable precision medicine. *Cancer Discov.* **8**, 1096–1111 (2018).

40. Pishvaian, M. J. *et al.* Overall survival in patients with pancreatic cancer receiving matched therapies following molecular profiling: a retrospective analysis of the Know Your Tumor registry trial. *Lancet Oncol.* **21**, 508–518 (2020).

41. Federico, A. Di *et al.* Hacking Pancreatic Cancer: Present and Future of Personalized Medicine. *Pharmaceuticals* **14**, 677 (2021).

42. NantKwest, ImmunityBio Announce Positive Interim Data on Survival Rates in Metastatic Pancreatic Cancer Trials | Business Wire. https://www.businesswire.com/news/home/20210113005928/en/NantKwest-ImmunityBio-Announce-Positive-Interim-Data-on-Survival-Rates-in-Metastatic-Pancreatic-Cancer-Trials.

43. X, P. *et al.* Application of natural killer cells in pancreatic cancer. *Oncol. Lett.* **22**, (2021).

44. Poulia, K. A. *et al.* Pancreatic Cancer and Cachexia—Metabolic Mechanisms and Novel Insights. *Nutrients* **12**, (2020).

45. Amon, R. *et al.* Directed Evolution of Therapeutic Antibodies Targeting Glycosylation in Cancer. *Cancers (Basel).* **12**, 2824 (2020).

46. Tumas, J., Tumiene, B., Jurkeviciene, J., Jasiunas, E. & Sileikis, A. Nutritional and immune impairments and their effects on outcomes in early pancreatic cancer patients undergoing pancreatoduodenectomy. *Clin. Nutr.* **39**, 3385–3394 (2020).

47. Perrone, V. G., Iacopi, S., Amorese, G. & Boggi, U. Impact of nutritional status on outcome of pancreatic resections for pancreatic cancer and periampullary tumors. *Hepatobiliary Surg. Nutr.* **9**, 669–672 (2020).

48. Qin, C. *et al.* Metabolism of pancreatic cancer: Paving the way to better anticancer strategies. *Mol. Cancer* **19**, 1–19 (2020).

49. Pezzilli, R. *et al.* Pancreatic enzyme replacement therapy in pancreatic cancer. *Cancers (Basel).* **12**, (2020).

50. Powell-Brett, S., de Liguori Carino, N. & Roberts, K. Understanding pancreatic exocrine insufficiency and replacement therapy in pancreatic cancer. *Eur. J. Surg. Oncol.* **47**, 539–544 (2021).

51. Iglesia, D. de la *et al.* Pancreatic exocrine insufficiency and pancreatic enzyme replacement therapy in patients with advanced pancreatic cancer: A systematic review and meta-analysis. *United Eur. Gastroenterol. J.* **8**, 1115–1125 (2020).

52. Bicakli, D. H., Uslu, R., Güney, S. C. & Coker, A. The Relationship Between Nutritional Status, Performance Status, and Survival Among Pancreatic Cancer Patients. *Nutr. Cancer* **72**, 202–208 (2020).

53. De La Iglesia-Garcia, D. *et al.* Increased risk of mortality associated with pancreatic exocrine insufficiency in patients with chronic pancreatitis. *J. Clin. Gastroenterol.* **52**, e63–e72 (2018).

2. Review of Target Audience’s Perceived Needs

In the Fall of 2021, we were proud to kick off Canada’s first accredited National Pancreas Conference in virtual format. It was a huge success by industry standards, featuring engaging sessions on impactful work in the field of diagnosis, treatment and management of pancreatic cancer. This year, we are proud to deliver the second National Pancreas Conference in person in Montreal, Quebec where we hope to foster a deeper collaborative network across the diverse array of disciplines and specialties that contribute to the field. In preparation for this year’s National Pancreas Conference, a focus group representing the target audience was surveyed to determine the perceived needs/interests of healthcare providers, researchers, and supportive care providers in the area of pancreatic cancer.
The needs assessment survey was well received with insight provided by 49 respondents. The target audience was well represented by the focus group, comprising 12 surgeons, 9 medical oncologists, 5 general practitioners, and 5 foundational researchers, as well as students, dietitians, radiation oncologists, nurses, and social workers, from seven different provinces across Canada (Figure 1).

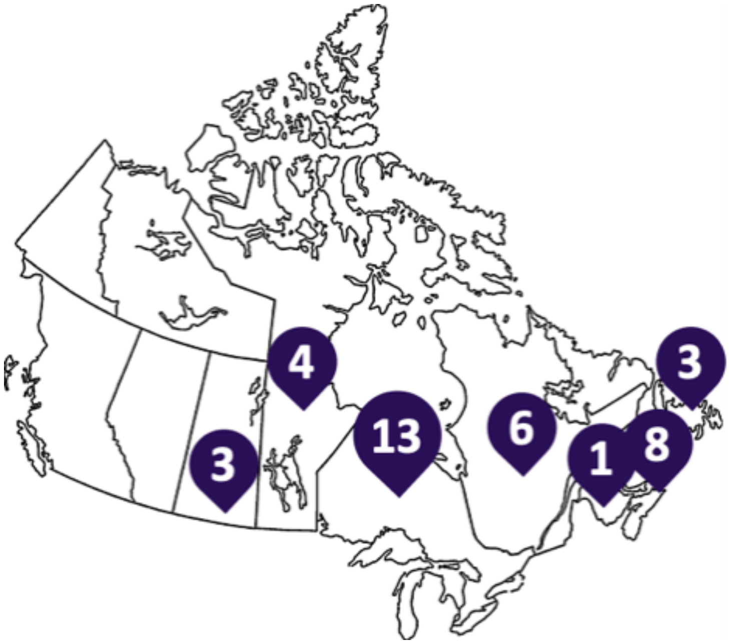

Figure 1. Illustration of geographical distribution of respondents across Canada.

Given the reduced COVID-related restrictions since the pilot NPC conference, it was of interest to determine our audience’s opinion towards moving to an in-person format. The survey responses revealed a 45% vote for a hybrid format, with the other 55% split evenly between virtual and completely in person (Figure 2). Based on this response, we concluded that a hybrid format would be most well received and optimize participation. We look forward to the opportunity to host in-person networking and social events that will encourage collaborative efforts across disciplines.

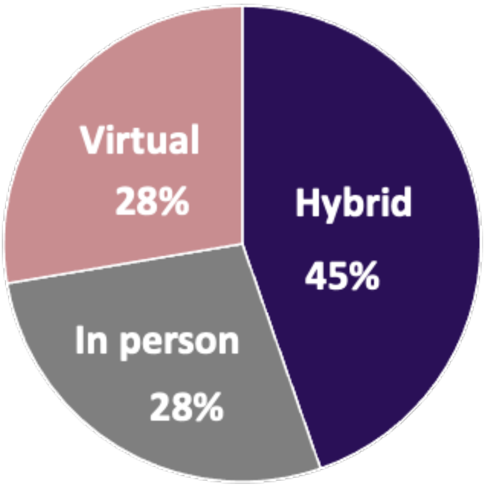

Figure 2. Pie chart of survey results regarding the desired format for the 2023 National Pancreas Conference.

A primary objective of conducting the focus group was to elucidate the topics that our target audience is most interested in gaining knowledge in. From this section of the survey, we note a general interest in a proactive and supportive care approach, with the top two responses being "Nutrition & Pancreatic Enzyme Replacement Therapy" and "Neoadjuvant Therapy". It has recently been shown by Dominguez-Munoz et al. that nutrition management and PERT provide equivalent improvement in overall survival to chemotherapy. And considering surgery exclusively offers the potential for a cure, the work being done in the neoadjuvant sector is critical, making surgical resection accessible to more patients. With that, *Advances in Surgery* was the third most requested topic with interest from 10% of respondents, followed by palliative/supportive care, immunotherapy, radiation, and racialized data (Figure 3).

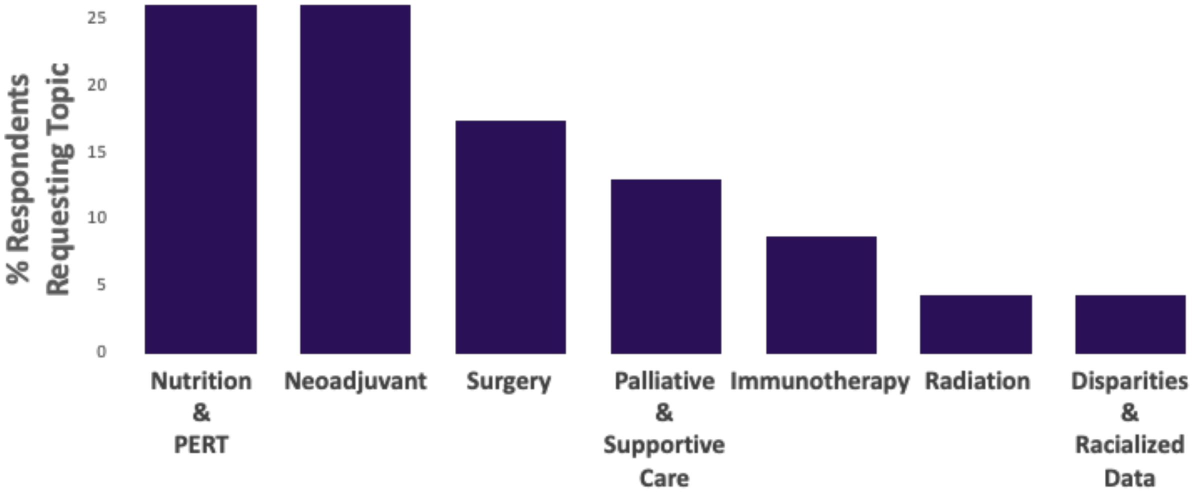

Figure 3. Bar graph summarizing the most frequently requested topics to be included as conference sessions.

Pancreatic cancer is a multi-faceted disease that requires multi-faceted treatment and management. It was therefore of interest to survey respondents for the referrals they make/recommend for localized and metastatic disease. Most referrals appear to be recommended with equal frequency for local and metastatic disease, however, referrals to surgical oncologists and gastroenterologists are biased towards localized disease[1]. Given the advancements in neoadjuvant therapy, making surgery an option for patients with "borderline resectable" disease[2,3], it is imperative for every patient to consult with a surgeon prior to committing to a particular course of treatment. Even if curative resection is not feasible, surgical staging can provide a more accurate prognosis and guide a more personalized treatment plan. It is optimistic to report that nutritional support/ PERT was the most frequently recommended referral, which supports the high interest in it as a session topic. It is puzzling to see that despite the advancements being made in precision medicine approaches, less than 50% of respondents are recommending genetic testing for either localized or metastatic disease. Likewise, with better outcomes reportedly being associated with clinical trial enrolment, it is surprising that only 60% of respondents recommended this referral. Overall, this portion of the needs assessment reflects some discrepancy between current literature and the referrals being made in practice, which emphasizes the need to encourage an interdisciplinary/ supportive care approach that benefits from all relevant sectors of healthcare to align with the most up-to-date translational research.

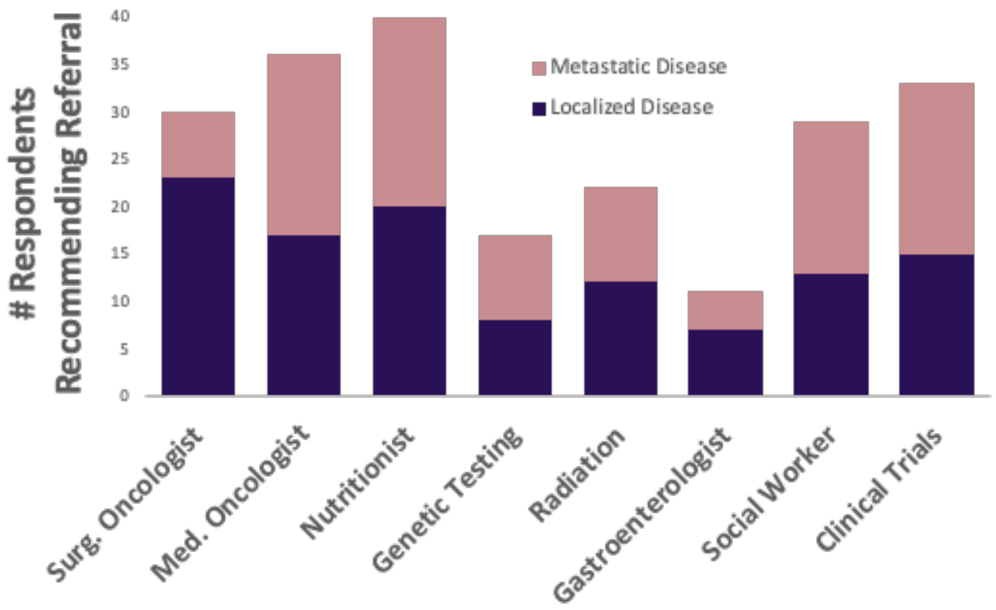

Figure 4. Bar graph profiling the types of specialists/care providers who are actively referring pancreatic cancer patients with metastatic vs. localized disease for pancreatic enzyme replacement therapy (PERT).

The 2022 National Pancreas Conference aims to host interactive sessions featuring nationally and world-renowned healthcare professionals and researchers in the areas of interest indicated by this needs assessment in combination with the unperceived needs reflected by a current review of recent literature. We will further facilitate interdisciplinary engagement by holding a poster exhibition wherein residents and junior researchers (i.e. graduate students) will

have the opportunity to present their most recent findings in an interactive and engaging setting. To encourage future collaborative efforts between disciplines of the healthcare field and research, we are currently accepting research proposals, up to three of which will be awarded a grant at a value of up to $100 000, on the condition that the work is either an interprovincial or interdisciplinary collaboration.
